# Climate change, phenology, and butterfly host plant utilization

**DOI:** 10.1007/s13280-014-0602-z

**Published:** 2015-01-09

**Authors:** Jose A. Navarro-Cano, Bengt Karlsson, Diana Posledovich, Tenna Toftegaard, Christer Wiklund, Johan Ehrlén, Karl Gotthard

**Affiliations:** 1 Department of Ecology, Environment and Plant Sciences, Stockholm University, 106 91 Stockholm, Sweden; 2 Department of Zoology, Stockholm University, 106 91 Stockholm, Sweden

**Keywords:** Brassicaceae, Diet width, Herbivory, Latitude, Lepidoptera, Species interactions

## Abstract

Knowledge of how species interactions are influenced by climate warming is paramount to understand current biodiversity changes. We review phenological changes of Swedish butterflies during the latest decades and explore potential climate effects on butterfly–host plant interactions using the Orange tip butterfly *Anthocharis cardamines* and its host plants as a model system. This butterfly has advanced its appearance dates substantially, and its mean flight date shows a positive correlation with latitude. We show that there is a large latitudinal variation in host use and that butterfly populations select plant individuals based on their flowering phenology. We conclude that *A. cardamines* is a phenological specialist but a host species generalist. This implies that thermal plasticity for spring development influences host utilization of the butterfly through effects on the phenological matching with its host plants. However, the host utilization strategy of *A. cardamines* appears to render it resilient to relatively large variation in climate.

**Electronic supplementary material:**

The online version of this article (doi:10.1007/s13280-014-0602-z) contains supplementary material, which is available to authorized users.

## Introduction

Climate change is considered one of the biggest threats to biodiversity today, and many species risk extinction due to a changed climate (Thomas et al. 2004; Parmesan 2006; Cahill et al. 2013). Species interactions make up an important part of biodiversity. Yet, knowledge of how such interactions are influenced by climate and habitat change is comparatively sparse (Lavergne et al. 2010). A change in climate or other environmental conditions may influence the strength of species interactions by relatively rapid plastic responses and by evolutionary changes over generations (Visser and Both 2005; Visser 2008; Altermatt 2010; Singer and Parmesan 2010). For example, if the phenology of an herbivore and its host plants in a seasonal environment is differentially influenced by temperature, a change in climate may lead to changes in the temporal overlap between the herbivore and its hosts (e.g., Singer and Parmesan 2010). As a result, the intensity of the interaction might change, or it may even disappear (Dewar and Watt 1992; Harrington et al. 1999). In herbivores using multiple hosts, climate change may lead to changes in the relative overlap with different hosts and thus to changes in host use. Such changes in interaction patterns are important to study as they influence both population dynamics and selection regimes, and are fundamental to understand how climate change might influence natural communities (Visser 2008).

A clear trend among many temperate species, including birds, plants and insects, during the past decades is that they have started to reproduce earlier during the spring and summer (Walther et al. 2002; Menzel et al. 2006; Parmesan 2007). Butterflies are temperature sensitive and all their life history stages are influenced by temperature (e.g., Dennis 1993; Karlsson and Wiklund 2005). Several studies have observed positive correlations between ambient temperatures during growth and development and date of the adult flight period, with an average advancement around 4 days/°C (Sparks and Yates 1997; Karlsson 2013). Some authors have also documented recent advancements in butterfly phenology in response to a warmer climate (Sparks and Yates 1997; Stefanescu et al. 2003; Menzel et al. 2006; Altermatt 2010; Diamond et al. 2011; Karlsson 2013).

However, recent comparative studies of butterflies in the UK (Diamond et al. 2011) and in Sweden (Karlsson 2013) reveal that shifts in phenology show a profound variation among species, making a more thorough inspection of the phenological responses justified. Previous studies have shown that variation in phenology shifts among butterfly species is associated with several life history traits, including overwintering stage, seasonal appearance, food plant species as well as several other factors, like food availability, habitat, altitude, and latitude (Altermatt 2010, 2012; Diamond et al. 2011; Illán et al. 2012; Karlsson 2013). For example, species overwintering as adults or as pupae tend to advance their phenology more than species overwintering as larvae or in their egg stage (Altermatt 2010; Diamond et al. 2011; Karlsson 2013).

Butterflies critically depend on plants as larval hosts and for nectar, and it is likely that optimal butterfly phenology in many cases strongly depends on the phenology of their host plants. Butterflies and host plants may respond differently to a warming climate, either because they use partly different cues or because their sensitivity to given cues differ (e.g., Menzel and Fabian 1999; Menzel et al. 2001; Parmesan 2007). Moreover, the direct effects of increased availability of CO_2_ may affect plant phenology more than the insects that use them as a resource. The relative importance of cues also varies among plants species (e.g., Rathcke and Lacey 1985), which may result in climate-dependent variation in relative abundances of different host species during the period of reproduction and growth of the butterflies (Schweiger et al. 2008). Such differences in reaction norms should lead to changes in species interactions with changes in climate.

Given that butterflies are strongly selected to maximize synchrony with their host plants and that host plants to some extent differ from each other and from butterflies in their response to increased temperatures, we expect butterfly responses to be related to the specific set of host plants that they depend on. For example, Diamond et al. (2011) showed that butterfly species with a small diet breadth, i.e., with only a few species of larval host plants, have higher advancement rates compared to species with a large repertoire of host plants. It can also be expected that butterfly species that feed exclusively on specific developmental stages of their hosts, e.g., flowers, young fruits, or young leaves, shift their phenology more strongly in response to warming than species that are not restricted to specific developmental stages.

An additional factor affecting plant and animal phenology is geographic location. The effects of latitude have been extensively scrutinized, and due to climate gradients stretching from south to north, growth and reproduction are generally occurring later in the northern parts (e.g., Myneni et al. 1997; Karlsen et al. 2007; Rötzer and Chmielewski 2001; Doi and Takahashi 2008). Butterflies show a relatively straightforward pattern with northern populations flying at later dates (Roy and Asher 2003; Karlsson 2013). However, not only phenology may vary along latitudinal gradients, but also the relative importance of different cues. Such differences would imply that plant populations of the same species along a latitudinal gradient respond differently to climate warming. This may lead to different responses among butterfly populations in order to maximize synchronization. Moreover, many butterfly species depend on multiple host plants, which use partly different environmental cues for start of development and that vary in relative abundance along latitudinal gradients. In combination, these relationships suggest that the realized pattern of host use will be affected by variation in climate, whether it is due to latitudinal differences or to long-term climate change. Such climate effects on host use are likely to be particularly important in butterfly species that are specializing on feeding on a specific phenological stage of their hosts. However, the effects of climate variation on patterns of host utilization in phenological specialists have rarely been studied. Indeed, detailed data on climate-induced changes of insect–host plant interactions over long periods of time are overall very rare (Visser and Both 2005; Singer and Parmesan 2010). One way forward is therefore to explore spatial variation in butterfly–host interactions along the climatic gradients of latitude or altitude.

Here, we review phenological changes in temperate butterflies over the last decades in Sweden and present results from an ongoing project exploring variation in the interaction between one phenological specialist, *Anthocharis cardamines*, and its multiple host plants along a latitudinal cline representing large variation in climate. More specifically we ask (1) How much has mean flight date changed in butterfly species in general, and in *A. cardamines* in particular, during the last 20 years in the same geographical area? (2) How well do temporal changes in mean flight dates for these species agree with the spatial trend along a latitudinal gradient? (3) To what degree do life history traits such as voltinism and overwintering stage correlate with changes in mean flight dates of butterflies in general? and (4) How does among- and within-species host plant use in *Anthocharis cardamines* differs along a latitudinal gradient?

## Materials and methods

### Study system

**Fig. 1:**
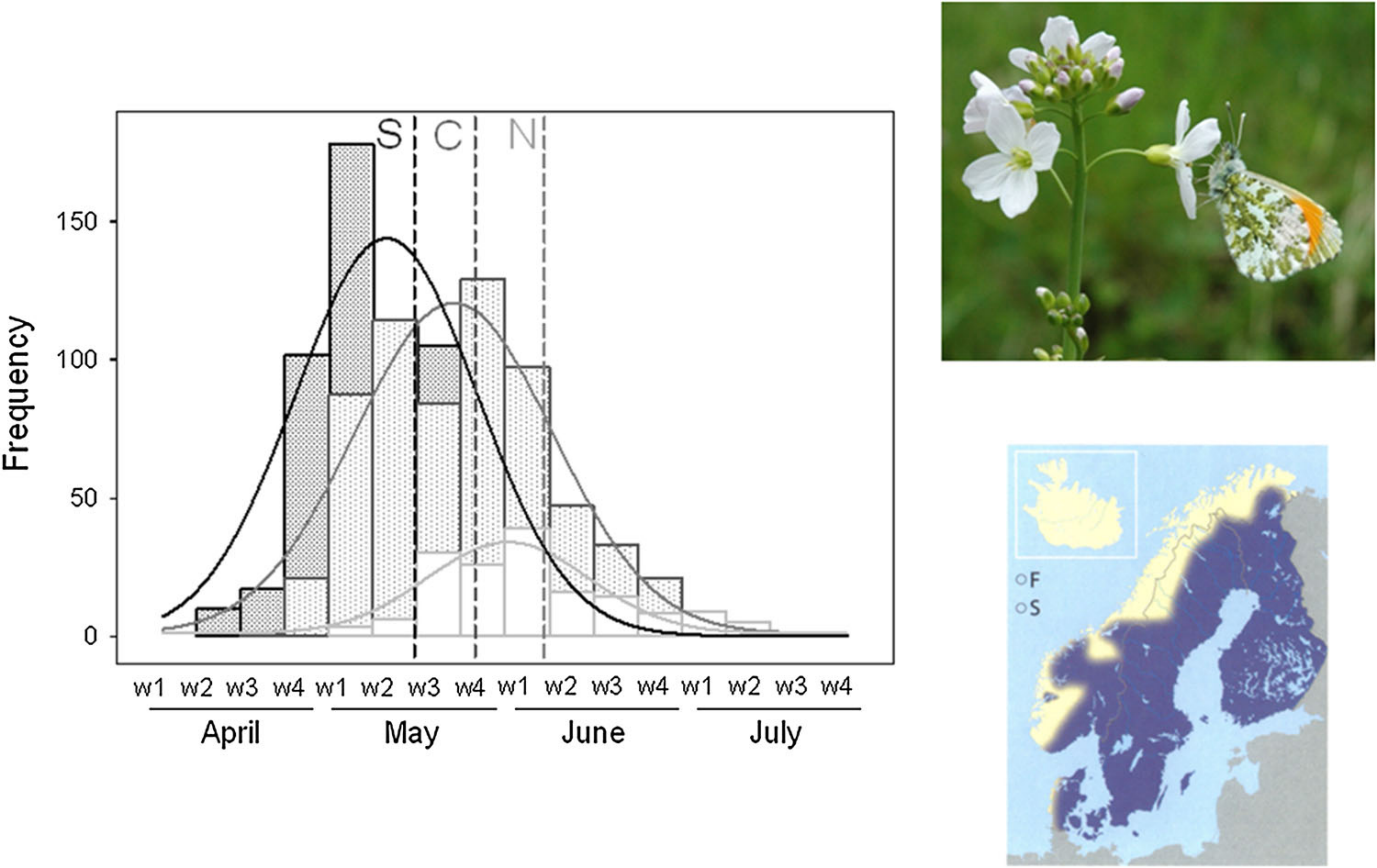
*Anthocharis cardamines* flying period and observation frequency (number of regional observations) in south (*black line*), central (*dark gray line*), and north (*gray line*) regions according to the 2010 “species gateway” data base (http://www.artportalen.se). Data were fitted to a Gaussian curve. *Vertical dashed lines* indicate the starting date of our samplings. Distribution map: Eliasson et al. (2005); Photograph by Christer Wiklund shows a male *A. cardamines* nectaring on *Cardamine pratensis*

The focal butterfly species in this study, the Orange-tip *Anthocharis cardamines* (Lepidoptera: Pieridae), and its host plant species constitute a particularly interesting model system to assess potential climate-dependent effects on host use. This butterfly is oligophagous on Brassicaceae using up to 17 different Brassicaceae species from 14 different genera within their range in Sweden (Wiklund and Åhrberg 1978; Arvanitis et al. 2007). The species is a phenological specialist in the sense that it feeds only on flowers and seedpods of its host, which are available for a period of approximately 1 month in spring at any given location in Sweden (Wiklund and Åhrberg 1978; Wiklund and Friberg 2009). As a result of its dependence of host plants that flower relatively early, *A. cardamines* flies early in the season (Fig. 1). Although both the butterfly and its host plants have a wide distribution in Europe, *A. cardamines* is obligatory univoltine and after larval development in spring, it pupates and enters diapause in early summer. Thus, the species spends most of the summer, and all of autumn and winter in the pupal stage.

### Changes in mean flight dates across butterfly species

We used the dataset compiled by Karlsson (2013) from the public database Swedish Species Gateway (http://www.artportalen.se) that contains observations of both amateur and professional naturalists. Using these data, we explore correlations between life history traits (voltinism, diapause stage) and temporal and latitudinal trends in phenology (mean flight date) of 66 butterfly species in Sweden and relate it to the special case of *A. cardamines.* For more detailed information about the data compilation, see Karlsson (2013).

### Study design latitudinal variation in *A. cardamines* host plant use

For our study of latitudinal variation in host plant use of *Anthocharis cardamines*, we included six host plant species: two perennial herbs: *Cardamine pratensis* L. and *Arabis hirsuta* (L.) Scop., one biennial: *A. glabra* (L.) Bernh., and three annuals: *Arabidopsis thaliana* (L.) Heynh., *Capsella bursa*-*pastoris* (L.) Medik., and *Thlaspi caerulescens* (J. Presl and C. Presl). *C. pratensis* grows on meadows, marshes, ditches, and stream margins (Arvanitis et al. 2007), whereas the other species use different habitats such as meadows, hillocks, rocks, and roadsides (Wiklund and Friberg 2009). *Arabidopsis thaliana* and *T. caerulescens* are the earliest species, flowering from March to April, and *A. glabra* and *C. pratensis* are the latest ones (June–July). *Capsella bursa*-*pastoris* has an extended flowering period (April to October) (Mossberg and Stenberg 2010). We distinguished between the tetraploid *C. pratensis* ssp. *pratensis* (hereafter, *C. pratensis*) and the octoploid *C. pratensis* ssp. *paludosa* (Knaf) Květ. (hereafter, *C. paludosa*), based on flower size and the type of cauline leaves (Arvanitis et al. 2007). These seven plant taxa span along the Swedish coast but their abundance varies from South to North (Mossberg and Stenberg 2010). A latitudinal delay in flowering from South to North within each species is expected as a consequence of the average monthly temperature, which is roughly correlated with the plant growing season (Sjörs 1999).

Data were collected between 17 May and 16 June 2010. We selected three regions ranging a 900 km S–N climatic gradient along the Eastern Swedish coast (Electronic Supplementary Material, Fig. 10.1007/s13280-014-0602-7): regions South (Scania Province; 55°49′N, 14°05′E), Centre (Uppland; 59°30′N, 18°35′E), and North (Ångermanland; 63°03′N, 18°19′E). We sampled consecutively in S (17–23 May), in C (28 May–3 June), and N (10–16 June). Within each region, 1–11 populations per host plant species were sampled in an area of approximately 50 km^2^ (Table 1).

**Table 1:** Host plant use in *Anthocharis cardamines*. The columns show for each species in each region: the number of populations sampled, the number of *A. cardamines* eggs found, the number of eggs per sampled plant (total number of eggs on a species in a region/total number of host plant individuals of this species within the region), the proportion of the total number of eggs laid on each host species, the number of plant individuals surveyed for eggs and, within brackets, the number in which phenotypic traits were measured. Missing data entries denote plant species not found in the respective regions

Region	Sampled populations	Host species	Number of *Anthocharis* eggs	Number of eggs/plants sampled	Regional proportional use of host plant (%)	Number of plants sampled
South Sweden	7	*A. thaliana*	10	0.009	9.1	1057 (445)
	–	*T. caerulescens*	–	–	–	–
	6	*C. bursa*-*pastoris*	6	0.007	5.4	795 (394)
	6	*C. pratensis*	30	0.012	27.3	2307 (587)
	4	*C. paludosa*	11	0.085	10.0	369 (63)
	11	*A. hirsuta*	53	0.032	48.2	1651 (645)
	–	*A. glabra*	–	–	–	–
Central Sweden	9	*A. thaliana*	3	0.004	1.5	815 (448)
	3	*T. caerulescens*	7	0.024	3.6	297 (177)
	8	*C. bursa*-*pastoris*	24	0.043	12.4	556 (331)
	3	*C. pratensis*	21	0.313	10.8	67 (67)
	9	*C. paludosa*	104	0.060	53.6	1746 (682)
	7	*A. hirsuta*	13	0.048	6.7	271 (264)
	2	*A. glabra*	22	0.057	11.3	386 (386)
North Sweden	4	*A. thaliana*	3	0.004	1.2	827 (415)
	7	*T. caerulescens*	13	0.004	5.3	3132 (365)
	3	*C. bursa*-*pastoris*	42	0.058	17.3	719 (156)
	1	*C. pratensis*	2	0.011	0.8	180 (177)
	4	*C. paludosa*	130	0.108	53.5	1208 (515)
	–	*A. hirsuta*	–	–	–	–
	1	*A. glabra*	53	0.757	21.8	70 (70)

We sampled data both at the level of plots and at the level of plant individuals. In each region, we searched randomly for occurrences of host plants. Whenever patches of one or multiple host plants were found, we established study plots. A plot was defined as the area covered by a patch of single or mixed host species populations, which was separated from the closest patch by at least 25 m. We judge that this design resulted in that differences in abundances among species in surveyed plots, roughly reflected abundances within the larger study region. At the plot level, we estimated the plot area including all the host plants in a patch as well as the total number of host plant individuals per species, yielding estimates of densities for each of the host plant species. Plot area ranged from 18 to 5382 m^2^. We searched all plants within plots for presence of butterfly eggs and estimated the mean number of eggs per plant within each plot as the total number of eggs divided by the number of plants individuals. At the individual level, we measured traits for a random subsample of the plants scored for each population. In populations with less than 100 plants, all plants were measured, whereas random samples of up to 150 plants were measured in larger populations. The measured traits were plant size (maximum shoot length), the total number of flowers on all shoots (total number of buds + flowers + pods at the time of recording), and the phenological state (number of pods divided by the total number of flowers at the time of recording). Overall, 16 453 plants were scored for egg presence at the plot level, and for 6187 of these, we also measured phenotypic traits (Table 1). Lastly, we used the database from the Swedish Species Gateway to assess how our sampling periods in S, C, and N regions were related to local butterfly phenology within each region.

### Statistical analyses

At the plot level, we used a generalized linear model (GLM) with Gaussian error structure and identity link function to study the effects of region and host species identity on the mean number of eggs per plant. Intra-specific host plant density was included in the model as a covariate. The mean number of eggs per plant was log-transformed, and its variation among regions and host species was examined with analysis of deviance. We also examined models including the summed density of all other potential host plant species in the plots. However, inter-specific host plant density had no significant (*P* = 0.25) effect on the mean number of eggs in a given species and was not included in the presented models.

At the individual level, we used GLMs with binomial error structure and logit link function to study the effects of host region, size, and phenology on egg presence (0 or 1). The two predictor variables, size and phenology, were the principal components PC1 and PC2, respectively, extracted from a principal component analysis (PCA) of the traits plant size, inflorescence size, and phenological state. We used PC1 and PC2 instead of the traits because original trait values were correlated (Pearson, *r* > 0.25, *P* ≤ 0.05). PC1 and PC2 explained 48.4 and 32.7% of the variance, respectively (accumulated explained variance = 81.1%). PC1 was positively correlated with the plant size and total number of flowers, whereas PC2 was correlated with the phenological state. PCA loadings for the three host plant traits are shown in Table 10.1007/s13280-014-0602-7 (Electronic Supplementary Material). As preliminary analyses detected significant interactions between region and traits for some species, we evaluated effects of trait variables in separate models for each region.

Mean ± SE in figures and tables is based on untransformed data. All GLMs were performed with R version 2.6.2 (R Core Team 2008). Multiple comparisons of means (Tukey contrasts) for the GLMs were made using the multcomp package (Hothorn et al. 2008). The PCA was carried out with SPSS 17.0 (SPSS Inc, Chicago, IL, U.S.A.).

## Results

### Correlations between life history traits and temporal and latitudinal trends

**Fig. 2:**
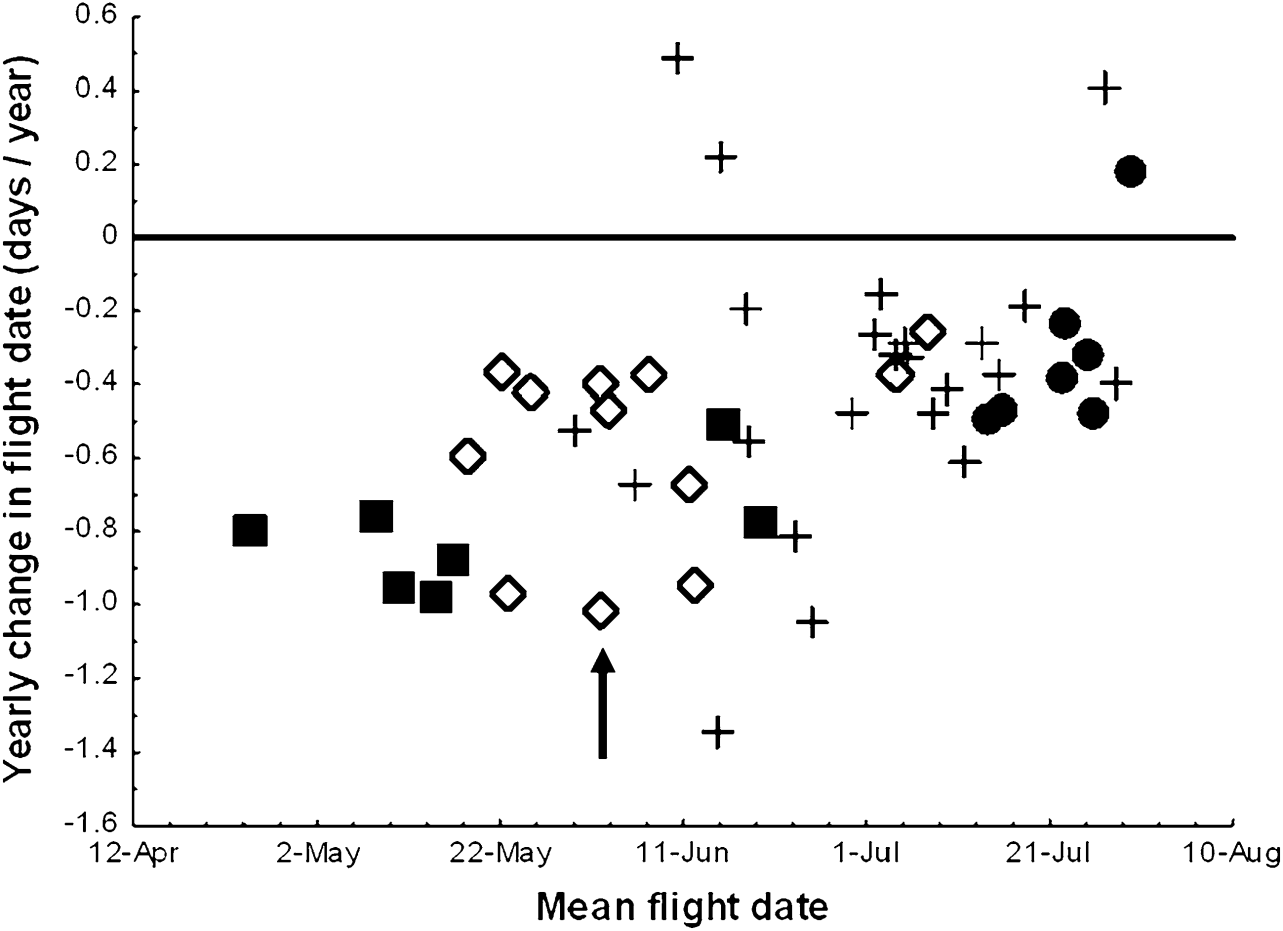
The relationship between mean flight date and yearly change in flight date in a set of butterfly species in Sweden during 1991–2010, *r* = 0.49, *P* < 0.001 (cf. Table 1 in Karlsson 2013), *symbols* represent overwintering stage; *squares* adult, *diamonds* pupal, *crosses* larval, and *dots* egg. The focal butterfly species, *Anthocharis cardamines*, in this study is marked with an *arrow*. The different overwintering stages differ significantly in respect to degree of yearly change in flight date, *F*(3,62) = 5.779, *P* = 0.0015. Redrawn from Karlsson (2013)

The average advancement of mean flight date of all 66 butterfly species was 0.36 days/year during the last two decades. Moreover, the mean flight date of the same investigated butterfly species showed a positive correlation with latitude (mean value is 1.20 days/degree of latitude). The advancement in mean flight date as well as the seasonal advancement at lower latitudes was both greater in *A. cardamines* than in the vast majority of other butterfly species in the region. It has advanced its mean flight dates during the last two decades with a mean value of 1.02 days/year, which is among the top three of all investigated butterfly species (Fig. 2). In addition, there are only 2 out of 66 species that show a steeper relationship between mean flight date and latitude than *A. cardamines* (3.41 days/degree of latitude).

**Fig. 3:**
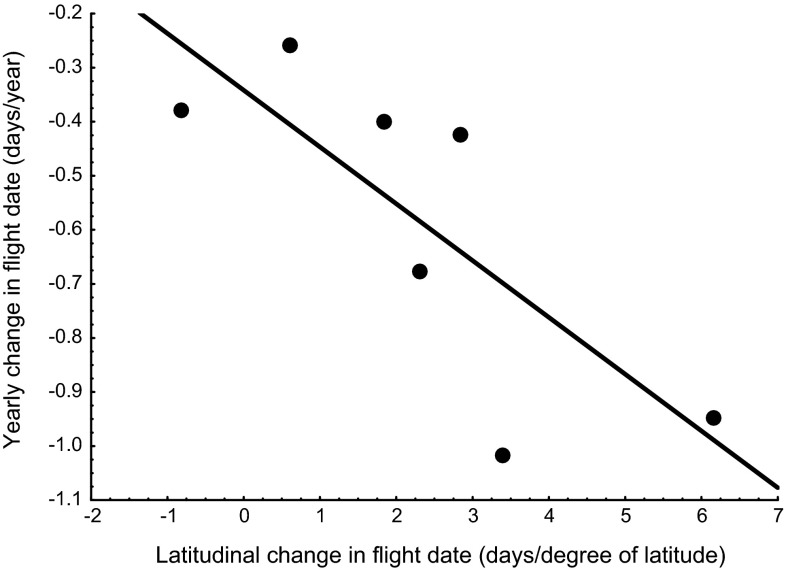
The relationship between yearly change in mean flight date and latitudinal change in mean flight date for all 7 species of butterflies in the dataset that overwinter as pupae and have an univoltine life cycle, *r* = −0.77, *P* = 0.04. *Anthocharis cardamines* is second from the right

Correlations between temporal and spatial trends were also evident in terms of a significant correlation between the yearly change in flight date and the dependence of flight date on latitude in the 66 species of butterflies investigated (*r* = −0.36, *P* = 0.015; cf. Fig. 5 in Karlsson 2013). In univoltine species overwintering as pupae, like *A. cardamines*, this relationship still holds true (Fig. 3). This suggests that spatial and temporal variations are partly caused by the same factors and that investigations of latitudinal trends should be useful to predict expected future temporal trends in butterfly species.

**Fig. 4:**
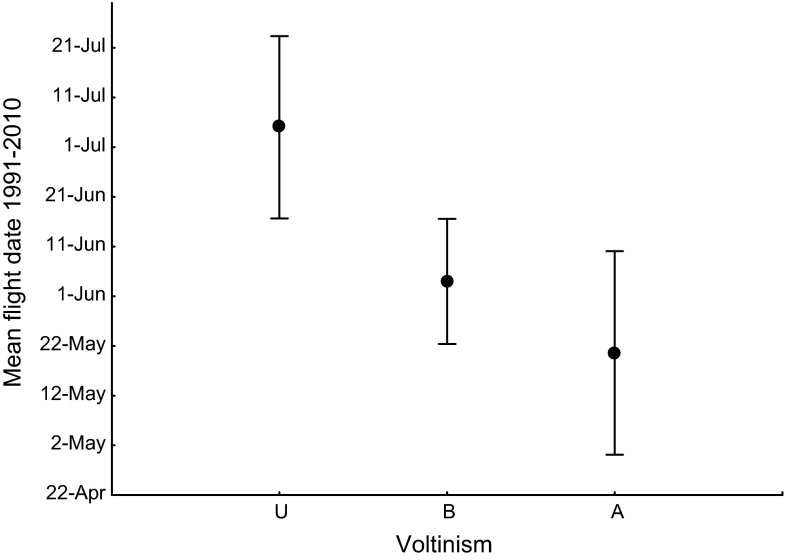
Comparison of mean flight date among univoltine (U, *n* = 46), bivoltine (B, *n* = 13), and adult overwintering (A, *n* = 7) butterfly species. Mean and SD, *F*(2,63) = 30.9, *P* < 0.001. Mean flight date is from Karlsson (2013), and overwintering stage is from Eliasson et al. (2005)

Since *A. cardamines* is a univoltine species throughout its geographic range, it is of interest to restrict the comparison to species sharing this characteristic. Among the 66 species investigated by Karlsson (2013), univoltine species generally have significantly later flight dates compared to bivoltine species (Fig. 4) (and also compared to adult overwintering species where adults appear two times per season but with generally only one cohort of larvae developing each year) (Fig. 3). *Anthocharis cardamines* has a relatively early flight also when compared only to other univoltine butterfly species; only 2 out of 46 investigated Swedish univoltine species fly at earlier dates than *A. cardamines*. To summarize, our focal species appears early in the season and much earlier at southern than at northern latitudes, and has advanced its flight dates in response to climate warming more strongly than most other butterfly species in the same area.

### Latitudinal variation in use of host plant species

A comparison with the records of *A. cardamines* during 2010 registered in the Swedish Species Gateway indicated that our census periods occurred 4–6 days after the peak flight period in all three regions (Fig. 1). Together with the results of previous studies showing that the egg stage in the field lasts 7–10 days (Wiklund and Åhrberg 1978) and that ca. 80% of the eggs are laid during the first half of the flight season (Wiklund and Friberg 2009), this strongly suggests that our census provided accurate assessments of host use in all regions.

**Fig. 5:**
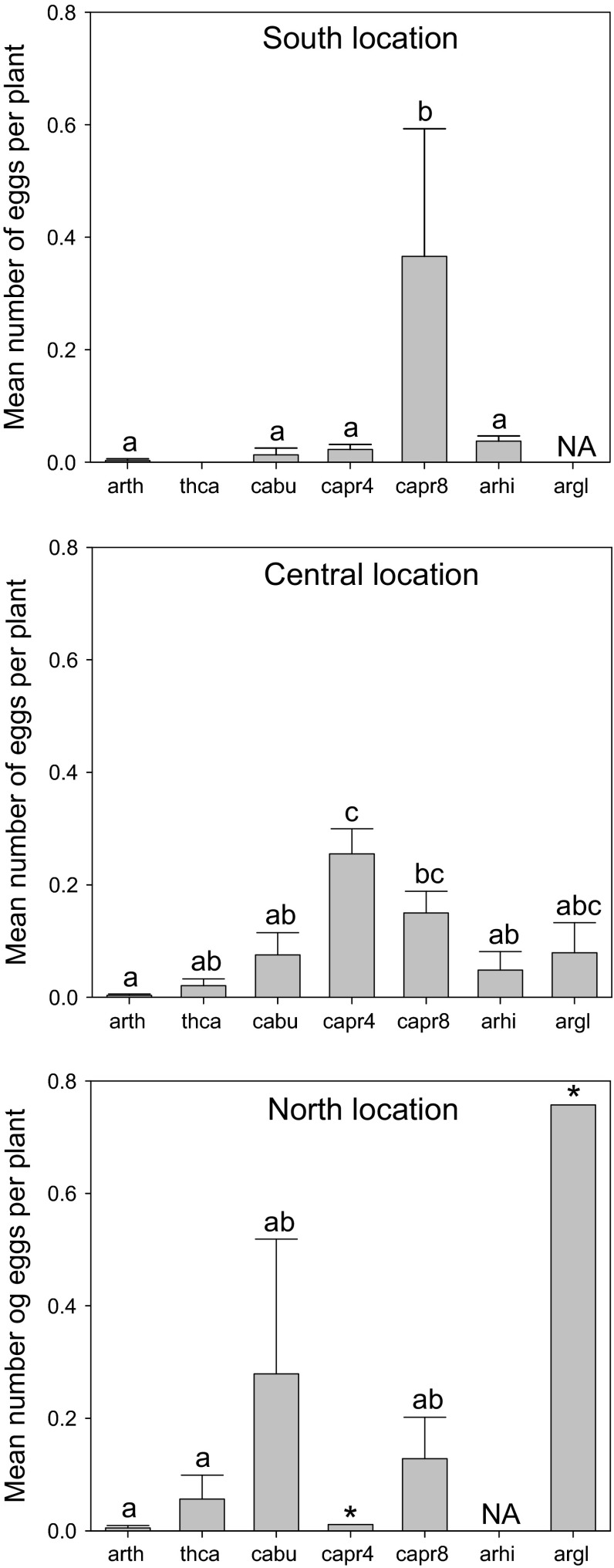
Mean number of eggs per plant (±SE) for seven different host plant species and three different regions along a latitudinal gradient. Means among species with different letter are significantly different (Tukey multiple Comparisons, *P* < 0.05). *NA* indicates that no populations of a host species were found in a region. The *asterisk* denotes that only one population was found. Species abbreviations: arth = *Arabidopsis thaliana*), thca = *Thlaspi* *caerulescens*, cabu = *Capsella* *bursa*-*pastoris*, capr4 = *Cardamine pratensis*), capr8 = *Cardamine paludosa*, arhi = *Arabis hirsuta*, and argl = *Arabis glabra*

The mean number of eggs per plant (number of eggs/number of plants in each plot) varied among host species (Table 2; Fig. 5). However, host use differed among the three regions (significant interaction region × species in Table 2). In the south region, the most used species for oviposition was *C. paludosa*, in the central region it was *C. pratensis*, and in the north region, *A. glabra* was the most attacked species (Fig. 5). From the butterfly’s perspective, there was a difference between regions concerning which host plant was most used for oviposition (Table 1).

**Table 2:** Effects of region, host species identity, and population density on the mean number of eggs per plant individual in each plot. Analysis of deviance with region and host species as factors and host plant density as a covariate

Source of variation	Number of eggs per plant		
	*df*	*F*	*P*
Region	2	1.816	0.274
Species	6	5.8058	**0.001**
Density	1	6.88	0.081
Region × species	9	3.983	**<0.001**

Effects significant at *P* < 0.05 are in bold

### Plant phenology and selection of host plants within species

**Fig. 6:**
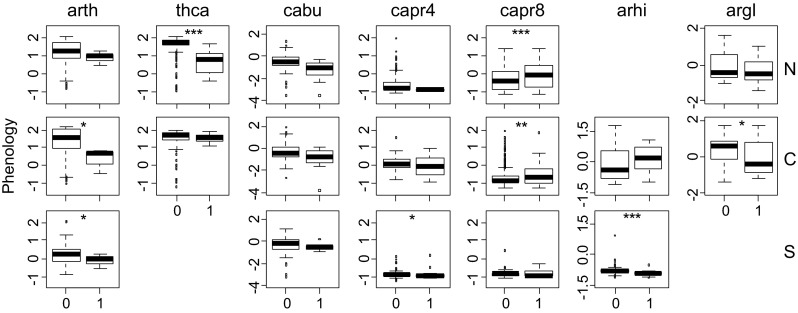
*Box-plots* showing the mean phenology (second axis from a PCA, see text) for plant individuals of seven different species and from three different regions that were either oviposited on by the butterfly *Anthocharis cardamines* (1) or that escaped attack (0). The seven host plant species were arth = *Arabidopsis thaliana*), thca = *Thlaspi caerulescens*, cabu = *Capsella bursa*-*pastoris*, capr4 = *Cardamine pratensis*), capr8 = *Cardamine paludosa*, arhi = *Arabis hirsuta*, and argl = *Arabis glabra*. The three regions were: south (S), central (C), and north (N). Significant differences between groups are indicated by *asterisks* (*P ≤ 0.05, **P ≤ 0.01, ***P ≤ 0.001). Note that scales differ among species

Among-individual differences in plant resistance to oviposition were related to phenology and size, but relationships differed among species and among regions (Fig. 6; Table 10.1007/s13280-014-0602-7 in Electronic Supplementary Material). Plants in more advance phenological stages were significantly more attacked in *A. thaliana*, *T. caerulescens*, *C. pratensis*, *A. hirsuta*, and *A. glabra*, while the opposite was true in *C. paludosa*. However, the effects of phenology significantly differed among regions for several species (significant effects of region × phenology in two species and of region × size × phenology in two additional species, Table 10.1007/s13280-014-0602-7). In *T. caerulescens*, late-flowering individuals were more attacked in the north, but there was no significant effect of phenology in the central region. In *C. pratensis*, late-flowering individuals were more attacked in the south region but there was no effect of phenology in the other regions. On average, butterflies preferred larger plants in all host species except for *T. caerulescens* (Fig. 10.1007/s13280-014-0602-7). However, in five of seven species, the effects of size differed along the latitudinal gradient (significant effects of region × size or region × size × phenology in Table 10.1007/s13280-014-0602-7). There were also significant effects of the interaction size × phenology in four of seven species.

## Discussion

There has been a general trend toward earlier flight periods in Swedish butterflies the last 20 years, and *Anthocharis cardamines* is among the species that has advanced its adult emergence most. Moreover, most Swedish butterfly species follow the typical pattern of later flight dates in more northern populations but this cline is steeper in *A. cardamines*. This type of correspondence appears to be a general trend as the rate of phenological change over time shows a significant correlation with the degree of change in flight date with latitude. This was true for both the full dataset with all Swedish butterflies as well as for the subgroup of univoltine, pupal diapausers, to which *A. cardamines* belongs. The results also show the quite intuitive pattern that butterfly species that are bivoltine start reproduction earlier in the year compared to univoltine species. This is most likely because selection in bivoltine species favors individuals that can use a longer period of the favorable season to produce two rather than one generation. In this respect, the early spring flight period of *A. cardamines* is clearly atypical for an univoltine butterfly in Sweden, occurring on average more than a month earlier than the other species (May 31 as compared to July 5). The early emergence of *A. cardamines* is very probably a direct consequence of that newly hatch larvae feeds on flowers and developing fruits of early flowering Brassicaceae plants.

During the last decades, there have been substantial phenological changes in a large number of animal and plant species (Walther et al. 2002; Menzel et al. 2006; Parmesan 2007). As the typical direction of change has been an advancement of phenological events, it has been causally linked to recent climate change and in particular the global increase in temperatures (Sparks and Yates 1997; Stefanescu et al. 2003). The results presented here add to this literature. More interestingly, this study and that of Karlsson (2013) found that the rate of phenological change over time was correlated with the phenological changes across latitudes. This suggests that species of butterflies that show strong latitudinal variation in phenology, presumably due to spatial variation in climate, also tend to show strong effects of changes in climate over time. This correspondence is expected if the adaptations that control butterfly life cycles and phenology include response to aspects of climate that changes in a similar way over time and space, and that populations along the latitudinal gradient respond in similar ways to climatic cues. While temperature is one obvious and important aspect of climate, other cues, such as the photoperiod, will not show this type of parallel change in time and space, i.e., the photoperiod at a given time of year varies with latitude while it is not influenced by temporal changes in climate at any given location. For our particular study system, this pattern suggests that it is reasonable to use the “space for time” paradigm to get a rough idea of how climate is likely to affect the phenology of *A. cardamines* and how this might influence its host utilization (Hodgson et al. 2011). Indeed, it seems likely that both temporal and spatial changes in the phenology of *A. cardamines* are reflecting strong effects of thermal conditions on the hatching of adults in comparison with other butterfly species. In support of this idea, the flight date of *A. cardamines* shows a strong response to ambient spring temperature during pupal development where an increase of 1°C advances flight date with 6.4 days. Mean value for other univoltine butterflies overwintering in the pupal stage is an advancement of 3.3 days/°C (cf. Karlsson 2013).

The regulation of life cycles of temperate insects is typically due to plasticity in relation to seasonal cues such as photoperiod and temperature (Tauber et al. 1986; Nylin and Gotthard 1998). Given the patterns shown here, it seems likely that the part of the life cycle determining adult emergence of *A. cardamines* in the spring is highly dependent on temperature. As this species spends the overwinter period in the pupal stage, it is the post-diapause pupal development in spring that will determine when the adults hatch. Hence, variation in adult emergence is likely to be strongly affected by the thermal reaction norms of pupal development. The advancement of spring phenology during the last decades as well as the latitudinal variation is likely to be largely a consequence of plasticity in response to variation in temperature (Gienapp et al. 2008; Merilä and Hendry 2014). However, thermal reaction norms have a genetic basis and may evolve in response to environmental changes. Indeed, recent experimental studies demonstrate that thermal reaction norms of post-diapause development in *A. cardamines* varies among populations from different latitudes suggesting that a part of the spatial variation in phenology seen here is due to local adaptation in these thermal reaction norms (Posledovich et al. 2014; Ståhandske et al. 2014). This also indicates that natural selection due to consistent directional change in climatic conditions over time will alter adaptations that are central for the evolution of phenology. From a climate change perspective, such evidence of local adaptation in thermal reaction norms suggests that responses to similar changes in temperatures will differ between regions along latitudinal gradients.

In the field survey examining host plant use of *A. cardamines* in three regions along a latitudinal gradient, we documented significant differences among regions in which of the host species that were used for oviposition. Given that the butterfly has strong preferences for plants in a given phenological stage (Arvanitis et al. 2008; this study), it is likely that effects of climate on the temporal overlap between the butterfly and each of the host plant species were important for these among-region differences. Such differences in temporal overlap between the butterfly and the different host plant species in response to latitudinal variation in temperature are to be expected if the thermal reaction norms differ between host plants and between the butterfly and its preferred host plants. It might seem reasonable to assume that phenological specialists, such as *A. cardamines*, are particularly sensitive to changes in climate. However, while the butterfly is expected to be under strong selection to match its phenology with the temporal distribution of Brassicaceae flowers in the spring, it is simultaneously strongly selected to be able to use multiple hosts given that the temporal overlap with one given species varies among years (Wiklund and Friberg 2009). As a result, the specialized feeding on the young fruits and seeds of its hosts is combined with the ability to utilize a quite wide host range of Brassicaceae species. Such a notion, that the species can be characterized as a phenological specialist but a host species generalist, is strongly supported not only by our data on latitudinal variation in host use but also by data on between-year variation in host use at a given site. During a 5-year study of the species at one locality in Sweden (the central location in this study), the species oviposited on 16 of the 18 available Brassicaceae species (Wiklund and Friberg 2009). A tentative conclusion is therefore that an assumed sensitivity of herbivores specializing in particular phenological stages of their host plants to climatic variation might sometimes be buffered by an ability to switch host plant species. If such host plant switching does not occur, we should expect very strong selection on consumer reaction norms to match the reaction norms of their resources.

Our results also show that within species, the phenological state and size of the hosts at the time of butterfly reproduction are important for oviposition. For most of the plant species, we found that later-flowering individuals attracted more eggs, although in one of the main hosts, *C. paludosa*, early flowering plants were significantly more used for oviposition. These results are important in two respects. First, they provide further evidence that phenological stage is important for butterfly host plant selection and that not only among-species choice but also choices within species are influenced by the phenological stage of the host plant. Moreover, several within-species patterns varied among regions suggesting that the exact temporal overlap between butterfly oviposition and host plant flowering had a strong effect on the realized host use across the climatic gradient described by the latitudinal range and that this overlap differed among regions. This suggests that the effect of climatic variation on host plant phenology, both in space and over time, will be of major importance for the realized host use of *A. cardamines.* Second, given that butterfly attack has strong negative effects on plant fitness (König 2014), the documented patterns of butterfly preferences translate to butterfly-mediated selection on plant flowering phenology. Given that butterfly attacks are relatively frequent in host plant populations, our documented patterns suggest that butterfly-mediated selection on plant flowering phenology may differ not only among different host plant species but also among regions within species.

## Conclusion

*Anthocharis cardamines* shows a strong phenological response to climatic variation compared to most other butterfly species that share its life history characteristics (univoltinism, pupal diapause). This pattern, in combination with it being a phenological specialist but a host species generalist, leads to substantial variation in host use both in time (Wiklund and Friberg 2009) and in space (this study). Unless the guild of its host plant species shows a very similar phenological alteration with the ongoing change in climate, which has been suggested for at least *Alliaria petiolata* and *Cardamine pratensis* in the UK (Sparks and Yates 1997), the realized host use of the butterfly is likely to be affected. However, the pattern of spatial variation in host use demonstrated here indicates that the species as a whole appears to harbor the necessary genetic variation, allowing it to respond both ecologically and evolutionarily to a relatively large range of climatic variation.

## Electronic supplementary material

Below is the link to the electronic supplementary material.

Supplementary material 1 (PDF 137 kb)
