# Supplementary material for: Climate change, phenology, and butterfly host plant utilization
Source: Ambio. 2015 Jan 9;44(Suppl 1):78–88. doi: 10.1007/s13280-014-0602-z (PMC4289000; doi:10.1007/s13280-014-0602-z)
Supplement: Supplementary file 1 — Supplementary material 1 (PDF 137 kb) [file 13280_2014_602_MOESM1_ESM.pdf]

Electronic Supplementary Material

Climate change, phenology and butterfly host plant utilization.

Jose A. Navarro-Cano, Bengt Karlsson, Diana Posledovich, Tenna Toftegaard, Christer Wiklund, Johan Ehrlén, Karl Gotthard

This supplementary material has not been peer reviewed.

Table S1 Principal component analysis (PCA) factor loadings for the three host plant traits measured to assess latitudinal variation in *A. cardamines* host plant use

	Rotated principal component	
	PC1	PC2
Phenological state	0.238	0.971
Plant size	0.838	-0.117
Inflorescence size	0.833	-0.160
Variance explained (%)	48.4	32.7

Table S2 Results of general linear models examining the effects of region, size and phenology, and their interactions on the probability of a plant individual being oviposited on (0 or 1) for seven host plant species. Effects significant at $P \leq 0.05$ are in bold

Species	Source of variation	Oviposition (Analysis of deviance)			
		df	*Resid. df*	Chi-square deviance	*P*
Arabidopsis thaliana	Region	2	1305	4.268	0.118
	Size	1	1304	49.598	**<0.001**
	Phenology	1	1303	1.711	0.191
	Region×Size	2	1301	8.859	**0.012**
	Region×Phenology	2	1299	0.096	0.953
	Size×Phenology	1	1298	10.817	**<0.001**
	Region×Size×Phenology	2	1296	5.460	0.065
Thlasphi caerulescens	Region	1	540	1.370	0.242
	Size	1	539	1.381	0.240
	Phenology	1	538	12.687	**<0.001**
	Region×Size	1	537	0.009	0.923
	Region×Phenology	1	536	9.189	**0.002**
	Size×Phenology	1	535	8.213	**0.004**
	Region×Size×Phenology	1	534	4.155	**0.042**
Capsella bursa-pastoris	Region	2	878	41.97	**<0.001**
	Size	1	877	41.50	**<0.001**
	Phenology	1	876	0.83	0.36
	Region×Size	2	874	3.37	0.19
	Region×Phenology	2	872	1.07	0.59
	Size×Phenology	1	871	14.06	**<0.001**
	Region×Size×Phenology	2	869	3.93	0.14
Cardamine pratensis	Region	2	828	55.55	**<0.001**
	Size	1	827	25.26	**<0.001**
	Phenology	1	826	4.55	**0.03**
	Region×Size	2	824	2.62	0.27
	Region×Phenology	2	822	7.82	**0.02**
	Size×Phenology	1	821	0.48	0.49
	Region×Size×Phenology	2	819	3.77	0.15
Cardamine paludosa	Region	2	1254	25.92	**<0.001**
	Size	1	1253	162.15	**<0.001**
	Phenology	1	1252	4.70	**0.03**
	Region×Size	2	1250	11.58	**0.003**
	Region×Phenology	2	1248	0.62	0.73
	Size×Phenology	1	1247	0.22	0.64
	Region×Size×Phenology	2	1245	5.00	0.08
Arabis hirsuta	Region	1	906	2.95	0.09
	Size	1	905	75.57	**<0.001**
	Phenology	1	904	1.99	0.16
	Region×Size	1	904	1.15	0.28
	Region×Phenology	1	903	2.83	0.09
	Size×Phenology	1	902	0.25	0.62
	Region×Size×Phenology	1	901	7.47	**0.010**

Arabis glabra	Region	1	454	47.110	**<0.001**
	Size	1	453	6.556	**0.010**
	Phenology	1	452	17.898	**<0.001**
	Region×Size	1	451	3.745	**0.053**
	Region×Phenology	1	450	1.758	0.185
	Size×Phenology	1	449	9.650	**0.002**
	Region×Size×Phenology	1	448	6.687	**0.010**

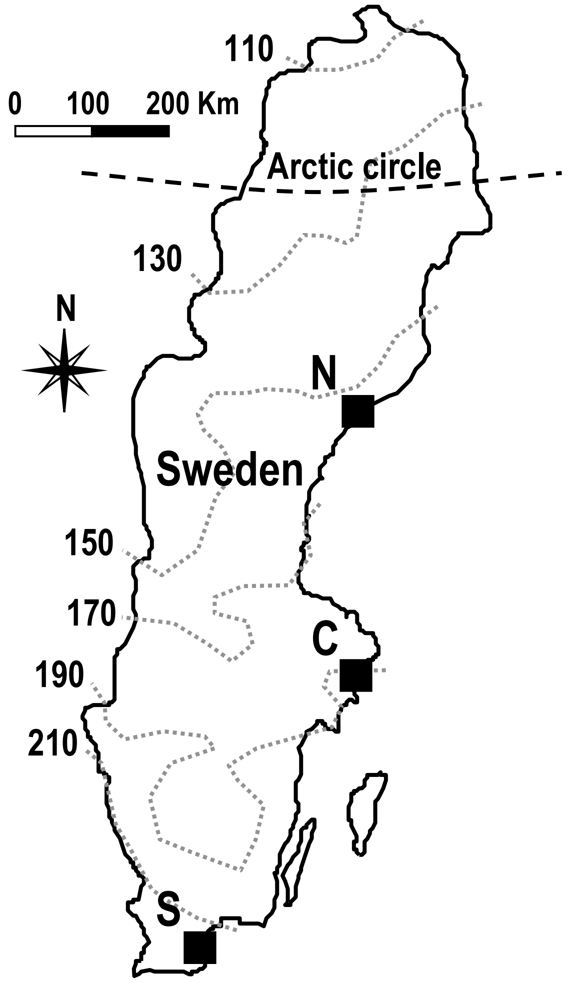

Fig S1 Sampling regions (south, central and north) along a south-north climatic gradient in Sweden and isoclines for the length of the climatic growing season in days (redrawn from Sjörs 1999).

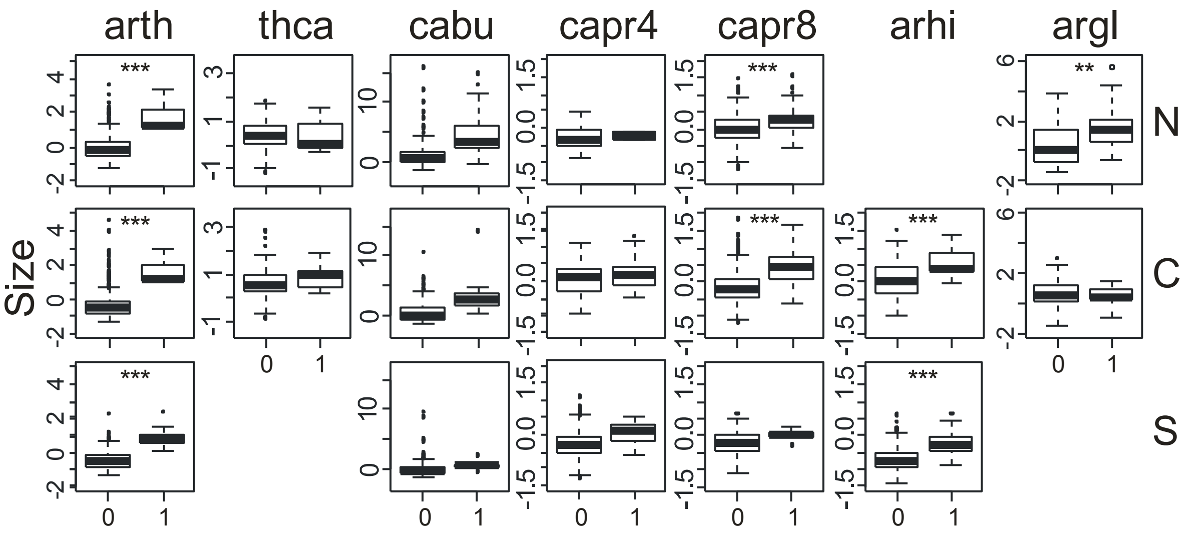

Fig. S2 Box-plots showing the effects of the predictor variable Size (PC1) on the oviposition (binary response variable; 0 = egg absence, 1 = presence) in the south (S), central (C) and north region (N). Significant effects of separate logistic regression models for the species with a significant interaction region × size or region × size × phenology are indicated by asterisks in the upper part of each box-plot (P ≤ 0.05*, P ≤ 0.01**, P ≤ 0.001***). Note the among-species different scale. Box-plots showing the mean size (first axis from a PCA, see text) for plant individuals of seven different species and from three different regions that were either oviposited on by the butterfly *Anthocharis cardamines* (1) or that escaped attack (0). The seven host plant species were: arth = Arabidopsis thaliana), thca = *Thlaspi caerulescens*, cabu = *Capsella bursa-pastoris*, capr4 = *Cardamine pratensis*), capr8 = *Cardamine paludosa*, arhi = *Arabis hirsuta*, and argl = *Arabis glabra*. The three regions were: south (S), central (C) and north (N). Significant differences between groups are indicated by asterisks (P ≤ 0.05*, P ≤ 0.01**, P ≤ 0.001***). Note that scales differ among species.
